# BMI and psychological distress in 68, 000 Swedish adults: a weak association when controlling for an age-gender combination

**DOI:** 10.1186/1471-2458-13-68

**Published:** 2013-01-24

**Authors:** Susanne Brandheim, Ulla Rantakeisu, Bengt Starrin

**Affiliations:** 1 Department of Social and Psychological Studies, Karlstad University, Karlstad, SE 651 88, Sweden

**Keywords:** BMI, Psychological distress, GHQ-12, Gender, Age

## Abstract

**Background:**

Study results concerning associations between body mass index (BMI) and psychological distress are conflicting. The purpose of this study was to describe the shape of the association between BMI and psychological distress in a large sample of Swedish adults.

**Methods:**

Data was measured with the General Health Questionnaire-12 (GHQ-12), in 68,311 adults aged 18–74. Self-reported data was derived from a merger of the 2000, 2004 and 2008 *Life and Health* (*Liv och Hälsa*) questionnaires focusing general perceived distress as well as living conditions. Logistic regression analysis was used to describe the association between BMI and psychological distress when controlled for age and gender in combination.

**Results:**

Women reported an overall higher psychological distress than men. A significant pattern of decreasing psychological distress with increasing age emerged among women in all BMI categories. Trends of this same pattern showed for men. Small or no differences were seen in psychological distress between those in normal weight, overweight, and obesity I categories (among women: 20.4%, 18.4%, 20.5%; among men: 12.8%, 11.2%, 12.9%). For both genders, any notable increase in psychological distress appeared first in the obesity II category (among women: 27.2%. Among men: 17.8%).

**Conclusions:**

Psychological distress decreases with increasing age regardless of BMI; a pattern more obvious for women. Being categorized with obesity II leads to a markedly higher psychological distress than being categorized with normal weight, overweight or obesity I. From this, we suggest that future obesity research focusing on psychological distress could investigate the role of stigma and norm susceptibility in relationships where people are evaluated through the eyes of the other.

## Background

In the medical model put forward by various medical and governmental health organizations, a body mass index (BMI) that is above normal is associated with a heightened risk of several diseases ([1,2]; World Health Organization, [3]). At the same time, a growing field of studies question whether BMI measurements reveal any general truth about an individual’s state of health [4-7].

The association between BMI and *psychological distress* is even less clear [8-15]. We follow scholars that view psychological distress as an emotional disturbance that may impact on as well as result from the social functioning and day-to-day living of individuals [16,17].

Studies on relationships between BMI and different measurements of psychological distress produced contradictory results. On the one hand, epidemiological studies have found positive correlations between BMI and psychological distress, although this was the case particularly in individuals with a BMI of 35 or higher [18,19]. BMI has also been associated with a diversity of psychological distress conditions such as low self-esteem, poor self-image, and depression; however, this association was significantly higher in individuals with a BMI above 40 [20].

On the other hand, Atlantis and Ball’s [21] population study, in which anxiety and depressive symptoms were the indicators of psychological distress, showed no association between BMI and psychological distress. Huang et al. [22] investigated a large population (n = 14,221) and found that BMI was associated to physical ill-health but not to psychological distress.

Several studies have found that various life factors influence the relationship between BMI and psychological distress. In an extensive review, Bacon and Aphramor [23] noted, however, that epidemiological studies rarely acknowledge factors like fitness, activity, nutrient intake, weight cycling, or socioeconomic status when considering the connection between BMI and psychological distress. When studies do control for these factors, increased risk of psychological distress disappears or is significantly reduced [24]. Lund et al. [25] found, for example, that unemployment among the morbidly obese affected their quality of life more than the weight did. In their study which controlled for individual’s overall health status, Loff and Crammond [26] found that the association between BMI and psychological distress disappeared.

While age is a factor that seems to affect the association between BMI and psychological distress, the extent to which it happens is unclear. Correlations have been found among the elderly [27,28]; however the studies did not control for their general state of health, such as chronic diseases and disabilities. Studies have also found that among the elderly being underweight because of an increase in general disease symptoms has a more negative impact on psychological distress than being overweight [29,30]. Some studies have found significant correlations between BMI and psychological distress among middle-aged women [31].

Several studies show that correlations between BMI and psychological distress seem to be stronger for women than for men [18]. However, there are also studies in which the results did not show gender differences regarding BMI and psychological distress [12,32].

Since studies of the relationship between BMI and psychological distress points in different directions, the relationship must be further investigated [22,33,34]. Overall, very few studies have investigated how age and gender in combination affect the association between BMI and psychological distress— especially in a general population. The aim of this study is to further explore the BMI and psychological distress association by taking age and gender into account.

## Method

### Data sample

This study was based upon data from three surveys carried out 2000, 2004, and 2008 in a mid-Swedish region (Liv och Hälsa 2000, 2004, 2008). The sample consisted of 203,918 individuals aged 18–84 years. The response rate was 63% (128,468 individuals). Only data for those in the age group 18–74 (*M* = 49 *SD* = 16) with a BMI 18.5 to 60 (*M* =26 *SD* =4.3) were selected. This final data sample consisted of 68,311 individuals.

The survey was approved by the boards of the County Councils of Uppsala, Sörmland, Västmanland, Värmland and Örebro. The survey was conducted under the jurisdiction of the Swedish law, the Helsinki declaration and international guidelines. An approval from an ethics committee was not applicable because the data are anonymous.

Individuals with values below BMI 18.5, which were considered underweight, were excluded from this study because the focus was on overweight and obesity in comparison to normal weight. Individuals with BMI above 60, which were considered extreme values, were also excluded. Individuals above 75 years of age were excluded. Very few of these individuals had BMI measurements indicating obesity. In addition, in this age group being underweight is held to be a greater health problem than being overweight [29,30].

### Instruments

The General Health Questionnaire 12 (GHQ 12) was used as a measure of psychological distress (Cronbach’s alpha = .90). Following Goldberg’s GHQ scoring method [35], the response alternatives were categorized as 0-0-1-1, which allows for a total score ranging from 0 to 12. A total score of three and above was categorized as psychological distress. The GHQ12 comprises questions about general level of happiness and symptoms of depression and anxiety symptoms over the last four weeks ([36] in [37]). The GHQ 12 avoids self-reflective bias as respondents are asked to value their *general* psychological distress independent of physical limitations.

We used the following four BMI classifications: normal weight (BMI 18.50–24. 99), overweight (BMI 25.00–29. 99), obesity I (BMI 30.00–34.99) and obesity II (BMI >35.00).

The sample was divided by age into six groups: 18–24, 25–34, 35–44, 45–54, 55–64, and 65–74. A weight-age variable was created by combining the four weight groups and the six age groups, resulting in 24 combinations according to the following scheme: 1 = Normal Weight/18–24 years, 2 = Normal Weight/25–34 years […], 24 = Obesity II/65–74 years.

### Processing of data

Statistical software employed for the analyses was SPSS 17.0. Conventional table analysis and logistic regression analysis were used to analyze data. Confidence Interval (CI) was set to 95%. Logistic regression analysis produces odds ratios to express a relative risk that an event or a condition will occur. In the logistic regressions the reference value is 1. We used the age group 65–74 years for our reference group in every BMI category.

## Results

As Table 1 shows, psychological distress decreases in relation to BMI for women and men in every age group when age and gender are taken into account except for a very small increase for men in the 25–34 years age group. This decrease is greater for women, ranging from 33.1% among the youngest to 10.1% in the oldest, compared with 17.1% in the youngest men to 6.7% among the oldest. Overall, women report a considerably greater psychological distress than men (20.0% compared to 12.2%). Women reported psychological distress ranging from 18.4 to 20.5% for the normal weight, overweight, and obesity I BMI categories. Men reported psychological distress from 11.2 to 12.9% for the same categories.

**Table 1:** Description of participants’ psychological distress (n = 68,311)

	**Women**			**Men**		
	***n***	**%**	**Psychological distress %**	***n***	**%**	**Psychological distress %**
	36.785	53.8	20.0	31.526	46.2	12.2
Age Groups						
18-24 y	3.496	9.5	33.1	2.435	7.7	17.1
25-34 y	5.509	15.0	27.6	3.847	12.2	17.7
35-44 y	6.129	16.7	24.3	4.746	15.1	16.5
45-54 y	6.687	18.2	20.5	5.262	16.7	13.9
55-64 y	7.607	20.7	14.2	7.013	22.2	9.8
65-74 y	7.357	20.0	10.1	8.223	26.1	6.7
BMI Categories						
Normal Weight 18.5-24.9	19.590	53.3	20.4	12.207	38.7	12.8
Overweight 25.0-29.9	11.635	31.6	18.4	14.585	46.3	11.2
Obesity I 30.0-34.9	4.106	11.2	20.5	3.793	12.0	12.9
Obesity II >35.0	1.454	3.9	27.2	940	3.0	17.8

For both genders, self-reported psychological distress makes a leap from the results in the previous category to those in the obesity II category (12.9 to 17.8% for men, 20.5 to 27.2% for women).

**Figure 1:**
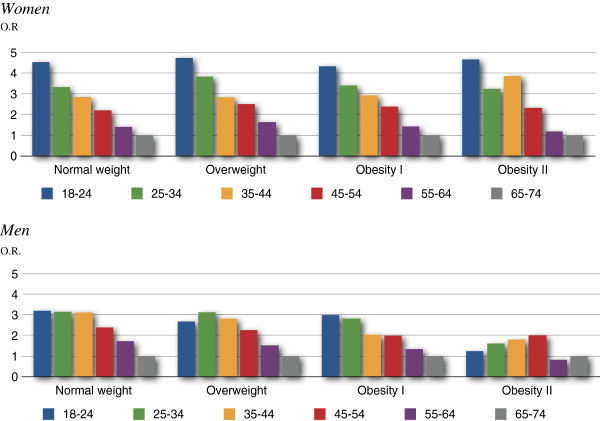
Psychological distress odds ratios within BMI category ordered by age groups women.

As Table 2 and Figure 1 show, the logistic regression analysis for psychological distress in relation to the 24 weight-age variables revealed a clear pattern of decreasing psychological distress with increasing age among women, in every BMI category.

**Table 2:** Logistic regression for psychological distress by BMI-age variable and gender

**BMI-Age**	**Women**			**Men**		
	**Psychological distress**			**Psychological distress**		
	***n***	**% (95% CI)**	**O.R. within BMI (*p*)**	***n***	**% (95% CI)**	**O.R. within BMI (*p*)**
**Normal W.**	19.590	20.4(19.9-20-9)	2.56	12.207	12.2(11.6-12.8)	2.43
**18.5-24.9**						
18-24y.	2.635	32.5(30.7-34.3)	4.53(*P < .001*)	1.690	17.5(15,7-19.3)	3.22(*P < .001*)
25-34y.	3.493	26.1(24.6-27.6)	3.32(*P < .001*)	1.894	17.2(15.5-18.9)	3.15(*P < .001*)
35-44y.	3.500	23.2(21.8-24.6)	2.84(*P < .001*)	1.731	17.0(15.2-18.8)	3.12(*P < .001*)
45-54y.	3.469	19.0(17.7-20.3)	2.20(*P < .001*)	1.790	13.6(12.0-15.2)	2.39(*P < .001*)
55-64y.	3.475	13.2(12.1-14.3)	1.44(*P < .001*)	2.314	10.2(9.0-11.4)	1.72(*P < .001*)
65-74y.	3.018	9.6(8.6-10.6)	1	2.788	6.2(5.3-7.1)	1
**Overweight**	11.635	18.4(17.7-19.1)	2.75	14.585	11.2(10.7-11.7)	2.24
**25.0-29.9**						
18-24y.	608	33.4(29.7-37.1)	4.73(*P < .001*)	578	15.2(12.3-18.1)	2.68(*P < .001*)
25-34y.	1.293	28.8(26.3-31.3)	3.83(*P < .001*)	1.450	17.4(15.5-19.3)	3.14(*P < .001*)
35-44y.	1.697	23.0(21.0-25.0)	2.83(*P < .001*)	2.268	15.8(14.3-17.3)	2.81(*P < .001*)
45-54y.	2.199	21.0(19.3-22.7)	2.50(*P < .001*)	2.633	13.2(11.9-14.5)	2.26(*P < .001*)
55-64y.	2.873	14.8(13.5-16.1)	1.63(*P < .001*)	3.564	9.3(8.4-10.2)	1.52(*P < .001*)
65-74y.	2.965	9.6(8.6-10.6)	1	4.092	6.3(5.6-7.0)	1
**Obesity I**	4.106	20.5(19.3-21.7)	2.58	3.794	12.9(11.9-13.9)	2.03
**30.0-34.9**						
18-24y.	178	36.0(29.0-43.0)	4.33(*P < .001*)	108	21.3(16.6-29.0)	2.99(*P < .001*)
25-34y.	477	30.6(26.5-34.7)	3.40(*P < .001*)	398	20.4(16.4-24.4)	2.82(*P < .001*)
35-44y.	658	27.5(24.1-30.9)	2.92(*P < .001*)	598	15.6(12.7-18.5)	2.04(*P < .001*)
45-54y.	745	23.6(20.6-26.6)	2.38(*P < .001*)	674	15.3(12.6-18.0)	1.99(*P < .001*)
55-64y.	951	15.6(13.3-17.9)	1.42(*P < .01*)	907	10.8(9.8-11.8)	1.34(*n.s.*)
65-74y.	1.097	11.5(9.6-13.4)	1	1.109	8.3(6.7-9.9)	1
**Obesity II**	1.454	27.2(24.9-29.5)	2.71	940	17.8(15.4-20.2)	1.41
**>35.0**						
18-24y.	75	44.0(32.8-55.2)	4.66(*P < .001*)	59	16.9(7.3-26.5)	1.24(*n.s*.)
25-34y.	246	35.4(29.4-41.4)	3.24(*P < .001*)	105	21.0(13.2-28.8)	1.61(*n.s*.)
35-44y.	274	39.4(33.6-45.2)	3.86(*P < .001*)	149	22.8(16.1-29.5)	1.80(*P < .05*)
45-54y.	274	28.1(22.8-33.4)	2.32(*P < .001*)	165	24.8(18.2-31.4)	2.01(*P < .05*)
55-64y.	308	16.6(12.4-20.8)	1.18(*n.s.*)	228	11.8(7.6-16.0)	.82(*n.s.*)
65-74y.	277	14.4(10.3-18.5)	1	234	14.1(9.6-18.6)	1

The only exception is for the 35–44 year age group in the obesity II category, where a rise of 4 percentage points is followed by an 11.3% drop in the 45–54 year age group. In Table 2, odds ratios for women show an almost identical pattern within the first three BMI categories. These gradient ratios are statistically significant. Figure 1 shows a step-like pattern of psychological distress in women by age group for the first three BMI categories.

As Table 2 and Figure 1 show, there is a similar trend for men in the first three BMI categories. A small exception is for 18–24 year olds in the overweight category, where self-reported psychological distress drops compared to the following age group. Values are statistically significant at the .001 level, except for the 55–64 year age group in the obesity I category (n.s.). As Figure 1 shows, there is a trend in men of decreasing psychological distress by increasing age in the normal weight, overweight, and obesity I categories.

In the obesity II category, the association with psychological distress changes is different for women than for men. For women, the strong pattern of decreasing psychological distress by increasing age remains, with the exception of an increase for the age group 35–44. Also, in this obesity II category, odds ratios for women in the 55–64 year age group were non-significant. Men in the obesity II category did not show a trend to decreasing psychological distress by increasing age. Instead, an increase in psychological distress in the first four age groups was followed by a drop in odds ratio in the 55–64 year age group.

## Discussion

For women, we found a clear step-like pattern of decreasing psychological distress with increasing age regardless of BMI, with a minor exception for obesity II. We have not been able to find this step-like pattern in any other study. Meanwhile, somewhat in line with our results, when dichotomizing age, Minniti et al. [38] found that older overweight and obese women had a better psychological status than their younger counterparts. The step-like pattern for men was similar to that of women, though less pronounced.

Regardless of BMI, women also reported a markedly higher psychological distress than men, which was noted in several other studies [18,22,27,39-41]. A possible explanation was put forward by Lim et al., ([39], who showed that, compared to men, women’s identity and self-image are to a larger extent connected to appearance and, thereby, also weight status.

Any notable increase of psychological distress in relation to BMI was seen first in the obesity II category. Several other studies have come to the conclusion that psychological distress rates are the same for normal weight and overweight persons [23,24,42-44]. What we did not expect, however in line with Knoesen et al. [45], was that this also included the obesity I category.

Possible methodological limitations exist in our study. In line with Smith (et al. [46]), we consider the General Health Questionnaire (GHQ-12) instrument a multi-dimensional and, hence, somewhat blunt measurement of psychological distress. Therefore our results should be viewed more as indications to be enhanced with the aid of more sophisticated instruments for measuring psychological distress. Another possible limitation was the small sized sample of men in the obesity II category (*n* = 940). This category contained not only the lowest number of respondents but also the widest spread in BMI, ranging from 35.00 to 60.00. This may have contributed to the non-significant results for psychological distress in men in the obesity II category.

A major strength of this study is the big sample size it employed. Here, we consider our results to call for future research on important cut-off points where body weight truly becomes a risk for increased psychological distress. Let us finally highlight an emerging hypothesis; one that deals with stigma and norm susceptibility.

Taking stigma into account, the mechanisms that alter the association between BMI and psychological distress at the top of the scale could be further explored by investigating the role of interpersonal relationships in which people are defined and evaluated through the eyes of the other. Deborah Carr and colleagues [47] have performed several studies on the stigmatization of obese persons. Their results show how obese persons that belong to social strata where obesity is less culturally normative are more likely to experience and perceive interpersonal mistreatment [47].

Perhaps a person’s distance to a cultural norm changes both that person’s exposure for and susceptibility to stigmatizing treatment –in turn affecting psychological distress. Considering some well-established normative claims of our self-producing society, where being male brings more advantages than being female, where being thin is more desirable than being fat and where being young is viewed as more attractive than being old, a norm /stigma susceptibility theory could help to explain the heightened psychological distress in the obesity II category.

## Conclusions

Psychological distress decreases with increasing age regardless of BMI. The pattern was obvious for women, less pronounced for men. Being categorized with obesity II, being woman or being younger lead to a markedly higher psychological distress than being man, older or categorized with normal weight, overweight or obesity I.

Future obesity research could investigate more thoroughly where and why an increasing weight becomes a risk for heightened psychological distress. Here we would suggest an exploration of the role of interpersonal relationships in which people are defined and evaluated through the eyes of the other, perhaps a study resting on the basis of a norm/stigma susceptibility theory.

## Competing interests

The authors declare that they have no competing interests.

## Authors’ contributions

SB initiated the study and held the overall responsibility. She designed the study, gathered background material, analyzed data, performed data base searches, prepared and completed the manuscript. UR performed data base searches, compiled parts of the background articles, revised the manuscript critically and made a draft to the introduction. BS continuously revised the design and the data analyses as well as the article’s intellectual content. All authors have read and approved the final manuscript.

## Pre-publication history

The pre-publication history for this paper can be accessed here:

http://www.biomedcentral.com/1471-2458/13/68/prepub
